# Tyrosine kinase inhibitors protect the salivary gland from radiation damage by increasing DNA double-strand break repair

**DOI:** 10.1016/j.jbc.2021.100401

**Published:** 2021-02-09

**Authors:** Trisiani Affandi, Angela M. Ohm, Dany Gaillard, Ami Haas, Mary E. Reyland

**Affiliations:** 1 Department of Craniofacial Biology, School of Dental Medicine, University of Colorado Anschutz Medical Campus, Aurora, Colorado, USA; 2 Department of Cell and Developmental Biology and the Rocky Mountain Taste and Smell Center, School of Medicine, University of Colorado Anschutz Medical Campus, Aurora, Colorado, USA

**Keywords:** radioprotection, DNA damage, DNA repair, salivary gland, protein kinase C-δ, tyrosine kinase inhibitor, DSB, double-stranded break, EGFR, epidermal growth factor receptor, HNC, head and neck cancer, HNSCC, head and neck squamous cell carcinoma, HR, homologous recombination, IMRT, intensity-modulated radiation therapy, IR, irradiation, NHEJ, nonhomologous end joining, TKI, tyrosine kinase inhibitor

## Abstract

We have previously shown that the tyrosine kinase inhibitors (TKIs) dasatinib and imatinib can protect salivary glands from irradiation (IR) damage without impacting tumor therapy. However, how they induce this protection is unknown. Here we show that TKIs mediate radioprotection by increasing the repair of DNA double-stranded breaks. DNA repair in IR-treated parotid cells, but not oral cancer cells, occurs more rapidly following pretreatment with imatinib or dasatinib and is accompanied by faster formation of DNA damage-induced foci. Similar results were observed in the parotid glands of mice pretreated with imatinib prior to IR, suggesting that TKIs “prime” cells for DNA repair. Mechanistically, we observed that TKIs increased IR-induced activation of DNA-PK, but not ATM. Pretreatment of parotid cells with the DNA-PK inhibitor NU7441 reversed the increase in DNA repair induced by TKIs. Reporter assays specific for homologous recombination (HR) or nonhomologous end joining (NHEJ) verified regulatation of both DNA repair pathways by imatinib. Moreover, TKIs also increased basal and IR-induced expression of genes associated with NHEJ (DNA ligase 4, Artemis, XLF) and HR (Rad50, Rad51 and BRCA1); depletion of DNA ligase 4 or BRCA1 reversed the increase in DNA repair mediated by TKIs. In addition, TKIs increased activation of the ERK survival pathway in parotid cells, and ERK was required for the increased survival of TKI-treated cells. Our studies demonstrate a dual mechanism by which TKIs provide radioprotection of the salivary gland tissues and support exploration of TKIs clinically in head and neck cancer patients undergoing IR therapy.

Most patients diagnosed with head and neck cancer (HNC) in the United States are treated with irradiation (IR) therapy alone or IR in combination with surgery or chemotherapy. While IR is targeted to the tumor, damage to nontumor adjacent tissues such as oral mucosa and the salivary glands also occurs, and in some cases can limit the course of therapy (1). This is despite improvements in radiation delivery such as intensity-modulated radiation therapy (IMRT), which enable more precise delivery of IR to the tumor (2). Permanent damage to the salivary glands occurs in up to 40% of HNC patients and can have a significant impact on quality of life, oral health, and nutrition (3). The free radical scavenger Amifostine is the only radioprotector currently available for sparing oral tissues, but it is not widely used due to systemic toxicity (4). This underscores the need for new radioprotective therapies that can prevent or mitigate IR damage to nontumor tissues without impacting tumor eradication.

Tyrosine kinases regulate many biological functions important for survival, including cell proliferation, cell motility, and angiogenesis and thus are important targets in cancer (5, 6). Imatinib and dasatinib are used clinically to treat leukemias and gastrointestinal stromal tumors (7, 8, 9, 10); however, neither has been shown to be effective against HNC in phase 2 clinical trials (11, 12). Conversely, studies from our lab and others demonstrate that dasatinib and imatinib can protect nontumor tissue in mice treated with IR or chemotherapy (13, 14, 15, 16). For example, dasatinib can protect against IR-induced intestinal injury in mice (14), and imatinib can protect mouse oocytes from cisplatin-induced cell death (13). Our studies show potent and durable radioprotection of salivary gland tissue and function *in vivo* when either TKI is delivered before or immediately after IR (16). TKIs mediate radioprotection of the salivary acinar tissues in part through suppression of apoptosis, suggesting that in this context tyrosine kinases are required for cell death (15, 16).

Given the paradoxical role of dasatinib and imatinib in suppressing apoptosis in normal tissues, but inducing cell death in some types of cancer, understanding the molecular basis for radioprotection by TKIs is critical. IR produces a wide variety of DNA lesions, with double-stranded breaks (DSBs) being the most abundant (17). DSB repair by nonhomologous end joining (NHEJ) or homologous recombination (HR) can increase cell survival and assure the genomic integrity of replicating cells. Here we have investigated the hypothesis that TKIs provide radioprotection by promoting the repair of IR-induced DNA DSBs. Given the complex nature of the tumor environment, our studies may have important implications both for radioprotection and for tumor therapy.

## Results

### TKIs accelerate repair of IR-induced DNA damage in salivary acinar cells

**Figure 1:**
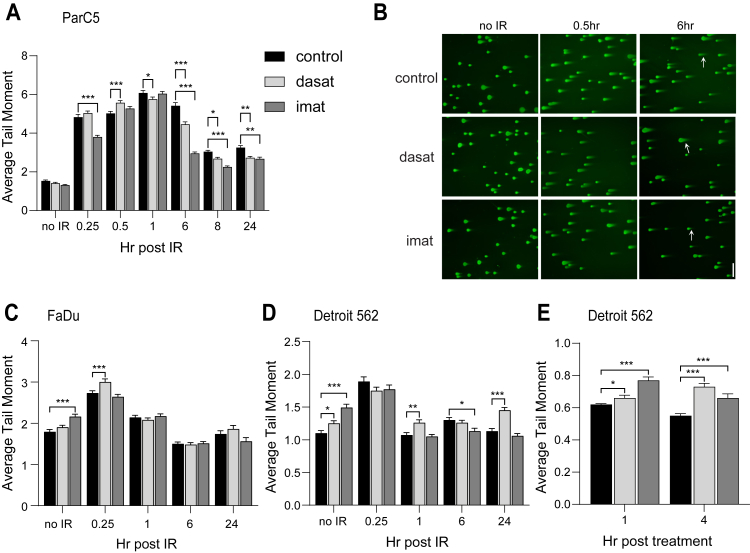
**TKIs accelerate repair of IR-induced DNA damage in ParC5 but not HNSCC cells***.* ParC5 (*A*), FaDu (*C*), or Detroit 562 (*D*) cells were treated with DMSO control (*black bars*), 50 nM dasatinib (*light gray bars*), or 10 μM imatinib (*dark gray bars*) for 30 min prior to 5 Gy IR exposure (legend in *A* is for all graphs). Following IR, cells were harvested at the indicated times and assessed for DNA damage using a neutral comet assay. *B*, Representative images from the comet assay in (*A*) at 10x objective, scale bars = 200 μm. *Arrows* indicate representative comet tails. *E*, Detroit 562 cells were treated with TKIs for the indicated times without exposure to IR. Statistics represent two-way ANOVA with Dunnett’s multiple comparisons within each time point or treatment to the corresponding DMSO control. ∗∗∗, *p* < 0.001; ∗∗, *p* < 0.002; ∗, *p* < 0.033. Shown is data from one experiment that was repeated three or more times.

We have previously shown that TKIs suppress apoptosis *in vitro* and provide robust radioprotection *in vivo* (15, 16). DSBs are the most frequent type of DNA lesions induced by IR, and their repair is essential for cell survival (17). To address the possibility that dasatinib and imatinib provide radioprotection by increasing DSB repair, we used a DNA comet assay to quantify residual DNA damage after IR, an indirect measurement of DNA repair. We show that pretreatment of ParC5 salivary acinar cells with either dasatinib or imatinib results in more rapid resolution of DNA breaks as compared with untreated cells (Fig. 1, *A* and *B*). At 6 h post IR, DNA damage is reduced 18% in dasatinib and 46% in imatinib-pretreated cells, compared with cells treated with IR alone (Fig. 1*A*). At 24 h post IR, DNA damage is reduced 16% in dasatinib and 18% in imatinib-pretreated cells compared with cells treated with IR alone. To verify that IR-induced DNA repair is not enhanced in TKI-treated HNC cells, we repeated the experiment described above using two head and neck squamous cell carcinoma (HNSCC) cell lines, FaDu and Detroit 562. Neither dasatinib nor imatinib increased repair of IR-induced DNA damage in either cell line (Fig. 1, *C* and *D*), in fact, DNA damage was increased following a 30-min pretreatment with TKIs alone, compared with control ParC5 cells (Fig. 1, *C* and *D*, no IR). Furthermore, treatment with either TKI alone for 4 h increased DNA damage up to 25% in Detroit 562 cells (Fig. 1*E*) consistent with a known effect of TKIs in inducing DNA damage in cancer cells (18). This supports our previous studies that show selectivity of TKIs for radioprotection of salivary gland acinar cells, without enhancing survival of HNSCC *in vitro* or *in vivo* (16).

**Figure 2:**
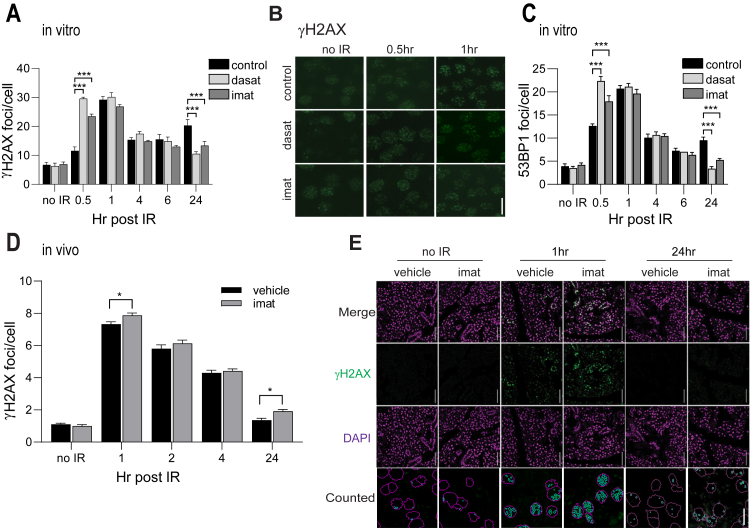
**TKIs accelerate repair of IR-induced DNA damage in salivary gland cells in vitro and in vivo.***A* and *C*, ParC5 cells plated on coverslips are treated as follows: DMSO control (*black bars*), 50 nM dasatinib (*light gray bars*), or 10 μM imatinib (*dark gray bars*) for 30 min prior to treatment with 1 Gy IR. Cells were harvested at indicated times post irradiation by fixation and stained for γH2AX (*A*) or 53BP1 (*C*) foci. *B*, Representative images from experiment in (*A*) showing γH2AX stained foci at 40x objective, *scale bar* = 20 μm. *D*, Immunohistochemistry for γH2AX foci was done on parotid glands from mice treated with or without imatinib, exposed to 10 Gy IR, and harvested at the indicated times. N=3–5 mice; 3–4 different fields of the parotid gland were analyzed per time point; n = 2 random optical z sections counted per imaged field. *E*, Representative pictures from quantification in (*D*) are individual 0.75 μm Z optical sections, *scale bars* = 50 μm. Images labeled “counted” show examples of foci (*green*) in cell nuclei (*magenta*) that were identified by the JQuantPro software, *scale bar* = 20 μm. Statistics represent two-way ANOVA with Dunnett’s (*A, C*) or Sidak’s (*D*) multiple comparisons within each time point to the corresponding control (*black bars*). ∗∗∗, *p* < 0.001; ∗∗, *p* < 0.002; ∗, *p* < 0.033.

We next investigated how TKIs impact DNA repair by quantifying IR-induction of γH2AX and 53BP1 foci, which represent sites of active DSB repair (19). In dasatinib or imatinib-pretreated cells, both γH2AX and 53BP1 foci increased much more rapidly after delivery of IR compared with control cells (Fig. 2, *A*–*C*). At 30 min after IR, γH2AX foci were increased 61% by dasatinib and 51% by imatinib (Fig. 2*A*), and 53BP1 foci were increased 44% by dasatinib and 30% by imatinib (Fig. 2*C*). Between 1 and 6 h post IR, γH2AX and 53BP1 foci numbers were similar in untreated and TKI-pretreated cells. However, at 24 h post IR, γH2AX foci were reduced 48% and 34%, and 53BP1 foci were reduced 64% and 44%, in dasatinib and imatinib-treated cells, respectively, compared with IR alone (Fig. 2, *A* and *C*). The faster formation of γH2AX and 53BP1 foci at early time points, and increased resolution at 24 h, indicates that repair of DSBs is more rapid and suggests that pretreatment with TKIs may “prime” salivary acinar cells for activation of DNA repair pathways. To determine if TKIs similarly increase IR-induced γH2AX foci *in vivo*, mice were pretreated with imatinib 1 h prior to delivery of 10 Gy IR and the parotid glands were harvested at various times post IR. IR results in a dramatic increase in γH2AX foci by 1 h, which was resolved by about 50% at 4 h (Fig. 2*D*). Similar to ParC5 cells *in vitro* (Fig. 2*A*), induction of γH2AX foci at 1 h was increased in mice pretreated with imatinib (Fig. 2, *D* and *E*), implying more rapid foci formation and accelerated DSB repair. At 24 h post IR γH2AX foci were reduced to nearly the level observed in no IR controls; however, an increase in γH2AX foci was still observed in mice pretreated with imatinib plus IR compared with IR alone (Fig. 2, *D* and *E*). Notably, γH2AX foci at this time point appeared more diffused as compared with the punctate foci seen at earlier time points (Fig. 2*E*). This could represent survival of cells with nonlethal DNA damage in TKI-treated salivary glands. However, whether diffuse γH2AX staining is always associated with DSBs is not clear. For instance, a recent study suggests that diffuse γH2AX staining after IR colocalizes with apoptotic cells and not with DNA damage (20).

### TKIs regulate double-stranded break repair

**Figure 3:**
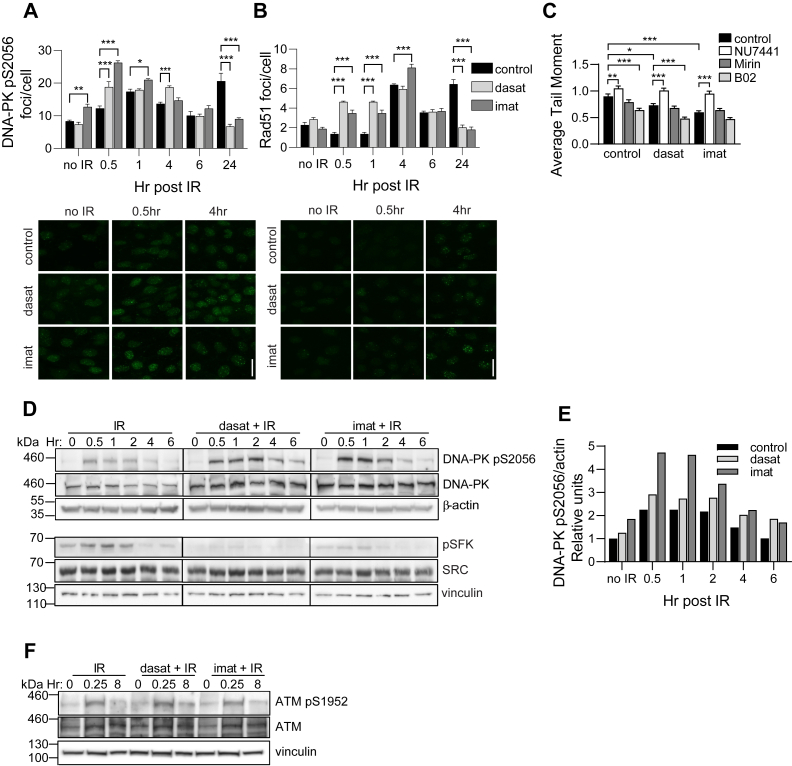
**TKIs regulate DNA repair via NHEJ and HR.***A* and *B*, ParC5 cells plated on coverslips are treated as follows: DMSO control (*black bars*), 50 nM dasatinib (*light gray bars*), or 10 μM imatinib (*dark gray bars*) for 30 min prior to treatment with 1 Gy IR (legend in *B* is for both *A* and *B*). Cells were harvested at indicated times post irradiation by fixation and stained for DNA-PK pS2056 (*A*) or Rad51 (*B*) foci. For *A* and *B*, representative fluorescent images are shown below the graphs at 40× objective, scale bars = 20 μm. *C*, ParC5 cells were treated with DMSO control, 50 nM dasatinib, or 10 μM imatinib (*black bars*), or in combination with 1 μM NU7441 (*white bars*), 25 μM Mirin (*dark gray bars*), or 20 μM B02 (*light gray bars*) for 30 min prior to 5 Gy IR. Cells were collected at 6 h post IR and assayed using a neutral comet assay. *D* and *F*, ParC5 cells were treated with DMSO control, 50 nM dasatinib, or 10 μM imatinib for 30 min prior to exposure to 5 Gy IR. Cells were harvested at indicated times (hr) post IR and immunoblotted with the indicated antibodies. Graph in (*E*) shows densitometry of the DNA-PK pS2056 blots in (*D*), normalized to β-actin control. All data shown are from representative experiments that were repeated at least three times. Statistics represent two-way ANOVA with Dunnett’s (*A* and *B*) or Tukey’s (*C*) multiple comparisons within each time point or treatment with the corresponding DMSO control (*black bars*) unless indicated by lines. ∗∗∗, *p* < 0.001; ∗∗, *p* < 0.002; ∗, *p* < 0.033.

DNA DSBs are repaired by HR in S and G2 phases of the cell cycle when a sister chromatid is available and by NHEJ throughout the cell cycle (21). To explore a role for dasatinib and imatinib in these two DSB repair pathways, we analyzed DNA damage foci immunostained for DNA-PK pS2056, an indicator of NHEJ pathway activation (Fig. 3*A*) (22), or Rad51, an indicator of HR pathway activation (Fig. 3*B*) (23). Treatment with either TKI resulted in more rapid and more robust formation of DNA-PK pS2056 foci, with peak formation at 30 min with TKIs versus 60 min in control (Fig. 3*A*). An increase in Rad51 foci was observed as early as 30 min in cells pretreated with TKIs, while Rad51 foci did not increase until 4 h in control cells, consistent with the known lag in HR repair relative to NHEJ (24). Significantly more Rad51 foci were present at 30 min, 1 h, and 4 h in TKI-pretreated cells compared with control cells, with formation of Rad51 foci maximal at 4 h for both groups (Fig. 3*B*). Our data indicates that treatment with TKIs accelerates DSB repair by both HR and NHEJ and results in less residual DNA damage at 24 h post IR. ParC5 cells were next pretreated with inhibitors of either NHEJ or HR in conjunction with TKIs. Treatment with NU7441, an inhibitor of DNA-PK (25), completely reversed the increase in DNA repair seen in cells treated with dasatinib or imatinib (Fig. 3*C*); however, neither Mirin, an inhibitor of Mre11-Rad50-Nbs1 (MRN) complex (26), nor B02, a Rad51 inhibitor (27), was able to reverse this increase (Fig. 3*C*). Interestingly, B02 actually further reduced DNA damage in control and TKI-pretreated cells, suggesting that inhibition of HR may activate alternative DNA repair pathways. This suggests that NHEJ may be the predominant pathway for DSB repair in ParC5 cells, consistent with studies that show at least 80% of IR-induced DSBs are repaired by NHEJ (17). However, an increase in Rad51 foci is also consistent with a role for HR in DSB repair (Fig. 3*B*). To explore this further, we asked if activation of upstream kinase regulators of NHEJ and HR is altered by TKIs. Dasatinib increased activation of DNA-PK 1.25-fold, while imatinib increased activation nearly 2.10-fold over IR alone as indicated by increased DNA-PK pS2056 at 30 min post IR (Fig. 3, *D* and *E*). No increase in ATM pS1952 (human ATM S1981) over control was detected with either TKI (Fig. 3*F*).

**Figure 4:**
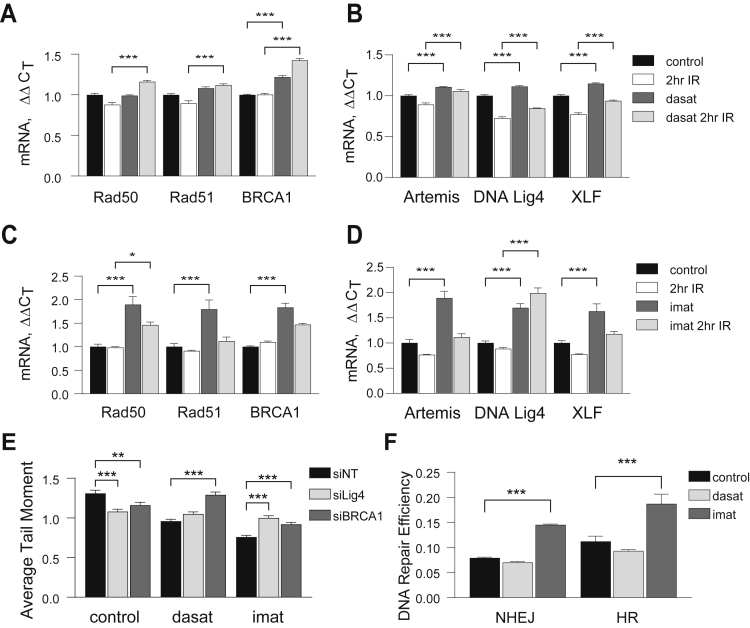
**TKIs regulate expression of genes required for DNA repair.***A–D*, Relative expression of HR (*A*, *C*) and NHEJ (*B*, *D*) genes was assayed by qRT-PCR. ParC5 cells were treated with DMSO control (*A–D*, *black* and *white bars*), 50 nM dasatinib (*A* and *B*, *dark gray* and *light gray bars*), or 10 μM imatinib (*C* and *D*, *dark gray* and *light gray bars*) for 30 min (see legend for *A* and *B*, *top*, or *C* and *D*, *bottom*). *Black* and *dark gray bars* in all graphs are untreated samples, while samples represented by *white* and *light gray bars* were treated with 5 Gy IR, and collected 2 h post IR. *E*, ParC5 cells transiently expressing nontargeting siRNA (siNT, *black bars*) or targeting rat DNA ligase 4 (siLig4, *light gray bars*) or BRCA1 (siBRCA1, *dark gray bars*) for 48 h were treated with DMSO, 50 nM dasatinib, or 10 μM imatinib for 30 min prior to exposure to 5 Gy IR. Cells were collected 6 h post IR and assayed using a neutral comet assay. *F*, Efficiency of NHEJ and HR repair pathways was analyzed using a fluorescent reporter assay. ParC5 reporter cells were treated with DMSO, 25 nM dasatinib, or 10 μM imatinib for 24 h prior to cotransfection with 5 μg SceI and 0.1 μg DsRed. Repair efficiency is expressed as the ratio of GFP+ to DsRed+. All data represent the mean ± SEM from three biological replicates. Statistics represent two-way ANOVA with Tukey’s (*A–E*) or Dunnett’s (*F*) multiple comparisons. Shown is data from one experiment that was repeated three or more times. ∗∗∗, *p* < 0.001; ∗∗, *p* < 0.002; ∗, *p* < 0.033.

To determine if expression of DNA repair genes is altered by imatinib or dasatinib, we analyzed expression of a subset of HR and NHEJ genes by qRT-PCR (Fig. 4, *A*–*D*). Basal expression of the HR genes BRCA1, and the NHEJ genes, Artemis, DNA Ligase 4, and XLF, is increased slightly by dasatinib pretreatment (up to 1.2 fold) (*black* versus *dark gray bars*, Fig. 4, *A* and *B*), while imatinib increased basal expression of all HR and NHEJ genes assayed 1.8- to 2-fold (Fig. 4, *C* and *D*). Surprisingly, in some cases IR resulted in a slight decrease in expression of HR and NHEJ specific mRNAs compared with control cells (*black* versus *white bars*, Fig. 4, *A*–*D*). BRCA1 was the mRNA most highly induced by dasatinib plus IR (1.4-fold over IR-only control, Fig. 4*A*) and DNA ligase 4 was the most highly induced by imatinib plus IR (>2-fold over IR-only control, Fig. 4*D*). Based on this data we investigated the contribution of BRCA1 and DNA ligase 4 to radioprotection by depleting each transcript using a siRNA pool. DNA ligase 4 and BRCA1 were depleted 81% and 70% respectively as determined by qRT-PCR (data not shown). Depletion of either DNA ligase 4 (siLig4) or BRCA1 (siBRCA1) in control cells slightly increased DNA repair after IR compared with cells expressing nontargeting siRNA (siNT) (Fig. 4*E*). However, in imatinib-pretreated cells, depletion of BRCA1 or DNA ligase 4 decreased DNA repair (Fig. 4*E*) while in dasatinib-pretreated cells only BRCA1 depletion decreased DNA repair. Finally, we utilized NHEJ and HR reporter assays (24) to ask if either TKI can increase repair at an HR or NHEJ-specific DSB (Fig. 4*F*). Imatinib increased repair at HR-specific lesions by 73% and repair at NHEJ-specific lesions by 87% (Fig. 4*F*). Surprisingly, pretreatment with dasatinib did not consistently increase repair at either lesion. This is consistent with data in Figures 3 and 4, *A–D* that shows a more robust effect of imatinib on DNA repair and expression of repair genes than dasatinib.

### Activation of ERK by TKIs contributes to enhanced cell survival

**Figure 5:**
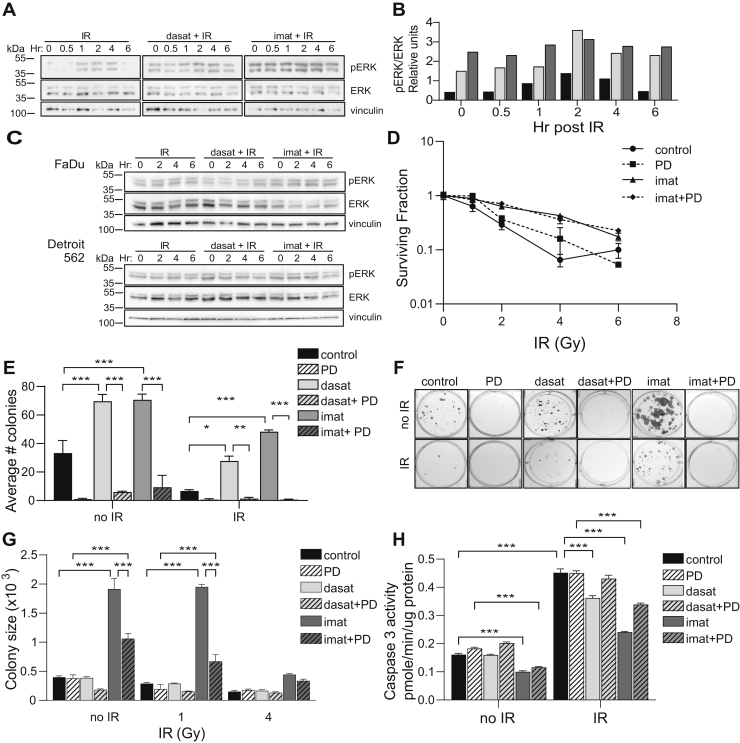
**Activation of ERK by dasatinib and imatinib contributes to enhanced survival.***A* and *B*, ParC5 cells were treated with DMSO control, 50 nM dasatinib, or 10 μM imatinib for 30 min prior to exposure to 5 Gy IR. Cells were harvested at indicated times (hr) post IR and immunoblotted with the indicated antibodies. Graph in (*B*) shows densitometry of the blots in (*A*), pERK normalized to total ERK and vinculin. *C*, FaDu (*top*) or Detroit 562 (*bottom*) cells were treated with DMSO, 50 nM dasatinib, or 10 μM imatinib for 30 min prior to 5 Gy IR. Cells were harvested at indicated times post IR (hr) and immunoblotted with the indicated antibodies. *D–G*, ParC5 cells were treated with DMSO control, 50 nM dasatinib, or 10 μM imatinib with or without PD98059 for 30 min and then left untreated (control) or treated with increasing doses of IR (*D* and *G*) or 4 Gy IR (*E* and *F*). Cells were trypsinized, counted, and plated in the presence of indicated inhibitors until visible colonies formed. *D*, Surviving fraction of colonies following IR dose response. *E*, Average number of colonies per condition following IR with representative images of quantification in (*F*). *G*, Quantification of colony size of 0, 1, and 4 Gy IR treatments from same experiment as in (*D*). *H*, Caspase 3 activity assay in ParC5 cells treated with DMSO (control), 50 nM dasatinib, 10 μM imatinib, 20 μM PD98059 for 30 min prior to 10 Gy IR and harvested at 48 h post IR. Data shown is from a representative experiment that was repeated three or more times. All data represent the mean ± SEM from three biological replicates. Statistics represent two-way ANOVA with Tukey’s multiple comparisons. ∗∗∗, *p* < 0.001; ∗∗, *p* < 0.002, ∗ *p* < 0.033.

Our previous studies suggested that TKIs can enhance tissue regeneration *in vivo* following IR (16). To address a potential prosurvival role for TKIs, ParC5 cells were pretreated with dasatinib or imatinib prior to IR delivery and activation of extracellular regulated kinase (ERK) was assayed. TKI pretreatment increased basal ERK activation in ParC5 cells 3- and 6-fold, respectively, and further activated ERK at all time points after IR (Fig. 5, *A* and *B*). In contrast, activated ERK was not increased by IR in FaDu or Detroit 562 HNSCC cells (Fig. 5*C*). To determine if ERK activation by TKIs increases survival of ParC5 cells after IR, we assayed clonogenic survival of cells treated with imatinib plus the MEK/ERK inhibitor, PD98059 (Fig. 5*D*). Imatinib dramatically increased the surviving fraction in response to IR; however, this appears to be largely independent of ERK as treatment with PD98059 did not significantly alter the surviving fraction at any IR dose (Fig. 5*D*). This suggests that ERK activation may be important for cell survival during plating and/or for colony proliferation. To address this, we quantified colony number (Fig. 5*E*) and size (Fig. 5*G*) in cells pretreated with either TKI, with or without PD98059 or IR treatment. Both TKIs increased total colony formation >2-fold in the absence of IR, consistent with increased survival during plating (Fig. 5*E*). After treatment with IR, colony formation in cells treated with dasatinib was 4-fold higher, while in cells treated with imatinib colony formation was increased 7-fold (Fig. 5*E*), suggesting an additional contribution of TKIs to IR-specific survival. Under all conditions colony formation was strictly dependent on ERK, suggesting that a major effect of ERK activation by TKIs is to increase plating efficiency. Likewise, colony size was increased dramatically by imatinib, but not dasatinib (Fig. 5*G*), and this was inhibited by PD98059. Cells in suspension can resist apoptosis by upregulation of survival pathways such as ERK (28). Given the dramatic effect of PD98059 on survival during plating, we asked if TKIs regulate apoptosis and if this is dependent on ERK activation. As shown in Figure 5*H*, both imatinib and dasatinib protect IR-treated cells from apoptosis, and this protection is dependent in part on ERK. Taken together, our data suggests that ERK activation by TKIs is important for cell survival during plating and that this may be mediated by TKI- and ERK-dependent suppression of apoptosis. Thus, in addition to increased DNA repair, ERK-dependent resistance to apoptosis likely contributes to radioprotection by TKIs.

## Discussion

Acutely radiosensitive tissues, including gastrointestinal mucosa and tissues in the oral cavity, are often unintended targets in patients undergoing IR therapy for cancer. While TKIs are widely used for cancer therapy, studies from our lab and others also show that some TKIs can protect against IR and chemotherapy-induced tissue damage (13, 14, 15, 16). We have shown that dasatinib, imatinib, and bosutinib can suppress IR-induced apoptosis *in vitro,* and that pretreatment of mice with dasatinib or imatinib provides potent and durable protection against IR-induced loss of salivary gland function (15, 16). Here we have investigated the mechanistic basis for radioprotection by TKIs. Our data indicates that both dasatinib and imatinib protect salivary gland function by increasing repair of IR-induced DSBs and by activation of ERK signaling through a mechanism that is selective for nontransformed cells.

A variety of approaches for radioprotection of the oral cavity are currently being explored, including delivery of free radical scavengers, treatment with growth factors and cytokines, and modulation of redox gene expression (3, 29). There are also concerted efforts underway to use salivary stem cells harvested prior to IR for salivary gland regeneration (30). Our lab has focused on inhibition of IR-induced apoptosis as a strategy for radioprotection of the salivary glands (31, 32, 33, 34). We show that TKIs function by inhibiting salivary acinar cell apoptosis immediately after IR and by promoting gland regeneration in the months after injury (16). A tissue-protective role for TKIs is supported by similar reports that show protection against IR-induced intestinal injury in mice (14), and cisplatin-induced cell death in mouse oocytes (13). It is not known if inhibition of other tyrosine kinases, particularly receptor tyrosine kinases (RTKs), can be tissue protective in some contexts. Epidermal Growth Factor Receptor (EGFR) has been studied extensively in this regard, with most studies showing that EGFR activation is associated with resistance to IR through a mechanism that involves an increase in NHEJ-directed DSB repair (35, 36). A likely target of dasatinib and imatinib in the context of apoptosis is PKCδ, an important regulator of genotoxin-induced cell death that is dependent on c-Abl and c-Src for activation (15, 31, 33, 37, 38). Notably, we have shown that activation of EGFR by DNA damaging agents induces DNA repair through a mechanism that is independent of PKCδ (39).

Imatinib was developed as an inhibitor of c-Abl, a tyrosine kinase that regulates a variety of biological functions relevant to the DNA damage response and apoptosis (40, 41, 42, 43). Dasatinib is a broad-spectrum inhibitor of non-RTKs, which primarily regulate cell proliferation and survival (44, 45); however, dasatinib can also inhibit c-Abl (6). Our current studies indicate that dasatinib and imatinib provide radioprotection in part by increasing repair of IR-induced genomic damage. IR-induced DSBs are primarily repaired by NHEJ, although HR also contributes when a DNA template is available (21). Highly error-prone forms of NHEJ, such as alternative end joining (alt-NHEJ) and single-strand annealing (SSA), can also contribute to repair under some circumstances (21, 46). c-Abl can both inhibit and activate DNA repair pathways, supporting a role in both cell survival and cell death (40, 43, 47). Activation of c-Abl often results in increased DNA instability, either through inhibition of DNA repair or by activation of SSA and alt-NHEJ-mediated DNA repair (48, 49, 50). In contrast, inhibition of c-Abl with imatinib can decrease SSA (51), inhibit inactivation of ATM/ATR signaling (52), and increase NHEJ (53). Thus, by suppressing SSA in favor of NHEJ and HR, TKIs may induce a less error-prone DNA repair, resulting in increased genomic integrity.

Our current studies show that TKIs induce more rapid and more complete repair of IR-induced DNA damage. We hypothesize that dasatinib or imatinib “primes” salivary acinar cells for DNA repair in part by increasing basal expression of mRNA transcripts required for NHEJ and HR (Fig. 4, *A*–*D*). This is supported by data that shows formation of HR and NHEJ-specific DNA damage foci occurs much more rapidly in TKI-pretreated cells (Fig. 3, *A* and *B*). Our studies suggest that imatinib and dasatinib have similar, but not identical, radioprotective effects perhaps reflecting divergence in activation of downstream signaling pathways. Overall, imatinib is a more robust inducer of all HR and NHEJ repair genes than dasatinib. Likewise, imatinib more robustly activates ERK and DNA-PK, and results in more rapid formation of DNA-PK pS2056 foci. Further, using a DNA repair reporter assay we show that imatinib (but not dasatinib) increases DNA repair by HR and NHEJ. We have confirmed increased formation of IR-induced γH2AX foci *in vivo* in mice pretreated with imatinib prior to IR. Although the increase in γH2AX foci in the imatinib-treated mice was only 1–2 foci per cell, based on our *in vitro* analysis, it is likely that this resulted from enhanced DNA repair.

We provide evidence that both TKIs can regulate HR and NHEJ; however, given that 80% of DSB repair typically occurs through NHEJ, this is likely the primary pathway regulated by TKIs (17). DNA-PK is an early DNA damage-sensing kinase and an important initiation factor in NHEJ. Addition of a DNA-PK inhibitor reverses protection by imatinib and dasatinib, and both TKIs increase activation of DNA-PK. Interestingly, treatment of ParC5 cells with the Rad51 inhibitor B02 actually decreased DNA damage, suggesting that inhibition of HR may drive cells to repair by an alternative pathway. Our data also shows that treatment with Mirin, an inhibitor of MRN complex, has no effect on TKI-regulated DNA repair, further indicating that NHEJ may be the main pathway regulated by TKIs (26). We identify DNA ligase 4 as important for DSB repair in imatinib-treated cells and show that BRCA1 is required for DNA repair in both dasatinib and imatinib-treated cells. Our finding that depletion of DNA ligase 4 has no effect on DSB repair in dasatinib-pretreated cells was surprising, as we also show that dasatinib regulates DNA-PK activation and that DNA-PK is required for increased DSB repair in cells treated with dasatinib. One possibility is that ParC5 cells depleted of DNA ligase 4 repair DSBs *via* an alternative NHEJ pathway. Masani and colleagues have shown that depletion of DNA ligase 4 in mouse B cells activates an alternative NHEJ pathway that is dependent on DNA ligase 1 and 3 (54).

Previously published data from our lab shows that TKIs can promote salivary gland regeneration after IR, suggesting that a pool of progenitor cells must remain, and that the genomic integrity of these cells is maintained (16). In addition to regulation of DNA repair, we show that both dasatinib and imatinib increase basal ERK activation through a mechanism selective for ParC5 cells. While ERK activation is required for the increased survival seen in cells treated with imatinib or dasatinib, we find that it is not required for IR-induced cell survival *per se*, but for survival during plating. ERK inhibition also increases apoptosis in TKI- and IR-treated cells. This is consistent with previous studies that show activation of ERK can protect cells from apoptosis during suspension (28). Likewise, we have previously shown that inhibition of ERK reduces colony formation (*i.e.*, plating efficiency) in non-small cell lung cancer cells by increasing apoptosis (55). In our current studies, imatinib, but not dasatinib, also dramatically increased colony size. This could be in part because imatinib is a more potent activator of ERK in the absence of IR. Importantly, our data suggest that dasatinib and imatinib do not provide radioprotection to HNC cells. In fact, both imatinib and dasatinib increased DNA damage in two HNC cell lines. This is consistent with previous data from our lab showing that neither TKI impacts IR treatment *in vivo* (16). Indeed, while both dasatinib and imatinib have been used widely in the clinic to treat specific solid and liquid cancers, neither has shown efficacy against HNC (11, 12).

Our studies provide mechanistic insights into previous studies from our lab that show TKIs provide robust and durable radioprotection of salivary gland function *in vivo* and that imatinib promotes salivary gland regeneration following irradiation (16). Here we have identified a role for TKIs in regulating DNA repair and show that ERK activation plays a role in TKI-mediated cell survival and cell proliferation. Increased repair of DNA damage, resistance to apoptosis, increased proliferation, and potentially other mechanisms are likely to contribute to preservation of salivary gland function and regeneration of salivary gland tissues *in vivo*. Taken together, our findings support further investigation of TKIs as a therapeutic strategy for protection of nontumor tissues in patients undergoing cancer therapy.

## Experimental procedures

### Cell culture

The ParC5 (RRID:CVCL_D695) cell line has been previously described (56). Detroit 562 (ATCC Cat# CCL-138, RRID:CVCL_1171) and FaDu (ATCC Cat# HTB-43, RRID:CVCL_1218) cells were obtained from the CU Anschutz Cell Technologies Shared Resource and cultured in DMEM/High glucose medium (Thermo Scientific, Logan, UT, USA, #SH30243.02) supplemented with 10% FBS (Sigma, St Louis, MO, USA, #F2442) and grown in 5% CO_2_ humid cell culture incubator. Cell line profiling for authentication was done through the CU Anschutz Cell Technologies Shared Resource at the University of Colorado Anschutz Medical Campus. Cells used in these experiments were within ten passages of authentication and were monitored for *Mycoplasma* once a month using the Plasmotest kit from Invivogen (San Diego, CA). For some experiments, subconfluent cells (60–80%) were treated with dasatinib (50 nM) or imatinib (10 μM) (Selleckchem, Houston, TX), or the DMSO vehicle, and irradiated using a Cesium-137 source. Other inhibitors used include NU7441 (DNA-PK), PD98059 (MEK1) (Selleckchem, Houston, TX), Mirin (Rad50/MRN), and B02 (Rad51) (Cayman Chemical, Ann Arbor, MI). The ON-TARGETplus siRNAs (SMARTpool) nontargeting control (siNT, D-001810-10-05) and targeting rat DNA ligase 4 (siLig4, L-089681-02-0005) and rat BRCA1 (siBRCA1, L-103578-02-0005) were purchased from Dharmacon-Horizon Discovery (Lafayette, CO).

### *In vivo* irradiation

C57Bl/6 (#00664) female mice were purchased from Jackson Laboratories (Bar Harbor, ME, USA). All animal procedures were performed in an AAALAC-accredited facility in compliance with the Guide for the Care and Use of Laboratory Animals, Animal Welfare Act and Public Health Service Policy, and were approved by the Institutional Animal Care and Use Committee at the University of Colorado Anschutz Medical Campus. Mice were housed in sterile ventilated cages with *ad libitum* access to irradiated food and water. Experiments started when mice reached 8 weeks of age. A single dose of 10 Gy IR was delivered to the head and neck using a Precision X-Ray X-RAD 225Cx irradiator at a dose rate of 2 Gy/min. Mice were anesthetized with 2% inhaled isoflurane (4% for anesthesia induction). Fluoroscopy scan was acquired for anatomical localization and radiation-treatment planning prior to IR. Imatinib (Imatinib mesylate, Cayman Chemical Cat #13139, 50 mg/kg, 5 mg/ml in sterile milliQ water) was given to mice *via* oral gavage 1 h before IR, and the salivary glands were harvested 1, 2, 4 h after IR. Unirradiated control mice were gavaged with 50 mg/kg imatinib or water 1 h before exposure to the same dose of isoflurane, and the salivary glands were harvested 4 h after anesthesia. Mice were euthanized by CO_2_ inhalation followed by cervical dislocation. The salivary glands were immersed in 2% paraformaldehyde overnight at +4 °C and then transferred to 20% sucrose in 0.1 M phosphate buffer overnight at +4 °C. Samples were embedded in O.C.T. Compound (Tissue-Tek 4583, Sakura Finetek, Torrance CA, USA), frozen on dry ice, and stored at –80 °C.

### Immunohistochemistry on tissue sections and image analysis

Immunostaining followed previously described procedures (57). Frozen 16 μm cryostat sections were collected on Superfrost Plus Slides (Fisher Scientific, Pittsburgh PA, USA). Immunoreactivity for each antigen was absent when primary antibodies were omitted. Sections were thawed, fixed in 4% PFA for 15 min at room temperature, rinsed in 1X phosphate-buffered saline, pH 7.3 (PBS), and placed on a heating plate at 37 °C for 5 min to improve adherence to the slide. Sections were then rehydrated in 1X PBS, and antigen retrieval was performed in 10 mM sodium citrate pH 6 + 0.05% tween20 at 95 °C for 15 min. After cooling on ice for 20 min in antigen retrieval solution, sections were incubated in blocking solution (5% normal goat serum, 1% bovine serum albumin, 0.3% Triton X100 in 1X PBS phosphate-buffered saline, pH 7.3) for 1 h at room temperature, and incubated with γH2AX (Cell Signaling, Cat # 9718S, RRID:AB_2118009) and Ecadherin (Developmental Studies Hybridoma Bank Cat # 5D3, RRID:AB_528116) antisera diluted 1/100 in blocking solution overnight at 4 °C. Sections were washed in 1X PBS, and blocking of endogenous avidin/biotin was performed with an Avidin/Biotin blocking kit (SP-2001, Vector Labs, USA). Sections were incubated with anti-rabbit biotin-conjugated antibody (Thermo Fisher Scientific, Cat # 65-6140, RRID:AB_2533969) diluted 1/500 in 1X PBS + 0.1% tween20 + 2.5% normal goat serum for 1 h at room temperature. Streptavidin-Alexa 488 (Thermo Fisher Scientific, Cat # S11223, RRID:AB_2336881) and goat anti-mouse IgG2a Alexa 555 (Thermo Fisher Scientific, Cat # A21137, RRID:AB_2535776) were diluted 1/800, in 1% bovine serum albumin + 0.3% Triton X100 in 1X PBS and then applied to the sections for 1 h at room temperature. Sections were counterstained with DAPI diluted 1/30,000 in 0.1 M phosphate buffer pH 7.2 for 3 min at room temperature, washed, and cover-slipped using Fluoromount G. Confocal fluorescence images were acquired using a Leica TCS SP8 laser-scanning confocal microscope and LAS X software driving a Dmi8 motorized microscope stand. Immunolabeled cells were analyzed on 0.75 μm optical sections. N=3–5 mice; 3–4 different ﬁelds of the parotid gland were analyzed per animal. Analysis of DNA damage γH2AX foci in the parotid gland was performed as described below on two optical Z sections, separated by at least ten sections, per field.

### Comet assay

Materials for the comet assay were purchased from Trevigen (Gaithersburg, MD). ParC5 and HNSCC subconfluent cells were treated with the indicated inhibitors (50 nM dasatinib, 10 μM imatinib, 1 μM NU7441, 25 μM Mirin, or 20 μM B02) or the DMSO vehicle and incubated at 37˚C for 0.5 h prior to exposing them to 5 Gy IR using a cesium-137 source. For the siRNA experiment, ParC5 cells were transfected with siNT, siLig4, or siBRCA1 at 25 nM using jetPRIME transfection reagent (Polyplus, New York, NY) and allowed to grow for 48 h prior to treatment with TKIs and IR. Following IR, cells were harvested at the indicated times for measurement of DNA damage using the neutral comet assay according to Trevigen Comet Assay protocol. Images were analyzed by Trevigen Comet Analysis Software (Version 1.3 days). DNA damage was quantified and expressed as mean tail moment.

### Analysis of DNA damage foci

Subconfluent ParC5 cells were grown on coverslips, treated with inhibitors or their respective vehicles, and exposed to 1 Gy IR. Cells were fixed in 2% paraformaldehyde in PBS at the indicated time post IR. For DNA-PK pS2056 and Rad51 foci staining, cells were permeabilized prior to fixing in 3% paraformaldehyde in PBS. Foci were detected by immunostaining at a dilution of 1:1000 with anti-γH2AX (Millipore Cat # 05-636, RRID:AB_309864), anti-53BP1 (Novus Cat # NB100-305, RRID:AB_10001695), 1:200 anti-DNA-PK pS2056 (Abcam Cat # ab18192, RRID:AB_869495), or 1:500 anti-Rad51 (Millipore Cat # ABE257, RRID:AB_10850319), followed by staining with secondary antibody Alexa Fluor 488 anti-mouse (Thermo Fisher Scientific Cat # A-11001, RRID:AB_2534069) or Alexa Fluor 568 anti-rabbit (Thermo Fisher Scientific Cat # A-11011, RRID:AB_143157) at dilution of 1:500. Coverslips were mounted on slides using Vectashield Vibrance mounting medium with DAPI stain (Vector Laboratories, Burlingame, CA, Cat # H-1800). Images were obtained on an Olympus BX51 fluorescent microscope with a 40x objective. A minimum of five fields were imaged per condition. JQuantPro software (kindly provided by Pavel Lobachevsky, Peter MacCallum Institute, Melbourne, AU) (58) was used to first identify nuclei based on the DAPI images to obtain a cell count. Object identification was inspected on each image to ensure splitting of single nuclei and accurate cell counts per field. Those objects were used as perimeters for identifying and counting foci number within the nuclei.

### Immunoblot analysis

Immunoblotting was done as previously described (15). Polyvinylidene difluoride membranes were stained with Ponceau S (Sigma-Aldrich, P3504) following transfer to confirm equal transfer and loading. Antibodies for DNA-PK-pS2056 (Cat # ab18192, RRID:AB_869495) and actin (Cat # ab49900, RRID:AB_867494) were obtained from Abcam (Cambridge, England). Antibodies for ERK 44/42 pT202/pY204 (Cat # 4370, RRID:AB_2315112), ERK (Cat # 4695, RRID:AB_390779), and vinculin (Cat # 13901, RRID:AB_2728768) were purchased from Cell Signaling Technology (Beverley, MA). DNA-PKcs (Cat # NB600-1203, RRID:AB_2170650) and ATM (Cat # NB100-309, RRID:AB_2243346) antibodies were purchased from Novus Biologicals (Littleton, CO). Antibodies for PKCδ (Cat # 14188-AP-1, RRID:AB_10638614) and ATM-pS1981 (Cat # AF1655, RRID:AB_2062935) were purchased from Proteintech (Rosemont, IL) and R&D Systems (Minneapolis, MN), respectively. Chemiluminescent images were captured digitally using the KwikQuant Image Analyzer (Kindle Biosciences, Greenwich, CT) and area density analysis performed using the KwikQuant analysis software.

### qRT-PCR

ParC5 cells were treated with inhibitors (50 nM dasatinib or 10 μM imatinib or DMSO control) for 0.5 h prior to exposure to 5 Gy IR. Cells were harvested at indicated times. Total RNA was purified using the Quick-RNA miniprep kit (Zymo Research), and concentrations were measured with a Nanodrop ND-1000 spectrophotometer (Nanodrop Technologies). cDNA was synthesized using Verso cDNA synthesis kit (Thermo Scientific). All qRT-PCR assays were PrimeTime qPCR Primer Assays purchased directly from IDT: Artemis (Dclre1c, Rn.PT.58.11997467), DNA ligase 4 (lig4, Rn.PT.58.34706582), XLF (Nhej1, Rn.PT.58.11756453), Rad50 (Rad50, Rn.PT.58.9875506), Rad51 (Rad51, Rn.PT.58.11260740), BRCA1 (Brca1, Rn.PT.58.8533844), rat GAPDH (forward: 5'-GCCAAATATGATGACATCAAGAAGG-3', reverse: 5’- GGTGTCGCTGTTGAAGTCAGAG-3’), Tfrc (Rn.PT.39a.22214841.g), B2m (Rn.PT.39a.22214834). RT-PCR measurements were done on a StepOnePlus Real-Time PCR Systems (Applied Biosystems) using SYBR Select Master Mix. The C_T_ was determined automatically by the instrument. Relative expression fold change was calculated using the 2^-ΔΔCT^ method (59). mRNA expression levels of nine housekeeping genes optimized for IR treatment (60, 61) were used as internal controls. Qbase+ software (Biogazelle) was used to determine the most stable reference gene(s) and to determine the number of genes needed to calculate the geometric mean (geNorm) used for normalization. GAPDH was determined to be a good reference gene for dasatinib-treated cells, while geNorm of B2M-1 and Trfc-1 genes was used for imatinib-treated cells. The relative expression levels were calculated as fold enrichment over the Ctrl 0 h cells (ΔΔC_T_). All samples were analyzed in biological triplicates and data are presented as mean ± SEM.

### Analysis of DSB repair using NHEJ and HR reporter lines

The NHEJ and HR reporter plasmids, pCBASceI (I-SceI expressing plasmid), and pDsRed2-N1 were kindly provided by Dr Vera Gorbunova (University of Rochester, NY). The experiment was done as previously described (24). To generate reporter cell lines, the linearized NHEJ or HR reporter cassettes (0.5 μg) were transfected into ParC5 cells using Amaxa Nucleofector 2B (Basic Epithelial Cells Kit, Program T-020, Lonza, Walkersville, MD). Post 24 h transfection, cells were selected with 100 μg/ml G418 for 7–10 days to generate stable ParC5-NHEJ and ParC5-HR reporter lines. For the DSB repair assay, the ParC5 reporter lines were pretreated with DMSO, 50 nM dasatinib, or 10 μM imatinib for 24 h prior to cotransfection with 5 μg I-SceI to induce DSBs and 0.1 μg pDsRed2-N1 to normalize for the differences in transfection efficiency. Cells were treated with DMSO, 50 nM dasatinib, or 10 μM imatinib and allowed to repair for 72 h. Cells were analyzed using a Gallios 561 flow cytometer (Beckman Coulter, Indianapolis, IN) and Kaluza software (Beckman Coulter). The repair efficiency was calculated by the ratio of GFP+ cells to DsRed+ cells.

### Clonogenic survival

ParC5 cells were treated with TKIs or PD98059 (Selleckchem, Houston, TX), singularly or in combination, for 30 min prior to irradiation with 0, 1, 2, 4, or 6 Gy IR. Immediately following irradiation, the cells were trypsinized, counted, and an equal number of cells (500 or 1000) were plated in triplicate wells per condition. Cells were plated in medium containing the same inhibitor combinations as the pretreatment conditions. Cells were allowed to grow for an additional 2–3 days at which time the media was replenished without addition of inhibitors. Cells were harvested once colonies were easily visualized and stained by fixation in 0.5% crystal violet stain containing 6% glutaraldehyde. Images were taken using the KwikQuant imager (Kindle Biosciences, Greenwich, CT) and number of colonies as well as colony size were quantified using ImageJ software (62). Surviving fractions were determined by determining the plating efficiency (PE) at 0 Gy for each treatment and calculating the surviving fraction as follows: SF = #colonies observed/(#colonies plated x PE).

### Caspase 3 activity assay

ParC5 cells were treated with TKIs or PD98059, singularly or in combination, for 30 min prior to irradiation with 10 Gy IR. Cells were then harvested post 24 h and assayed for caspase 3 activity with the Caspase-3 Cellular Activity Assay Kit PLUS (Biomol, Farmingdale, NY), which uses N-acetyl-DEVD-p-nitroaniline as a substrate, according to the manufacturer’s instructions.

### Statistical analysis

Data shown in figures are representative experiments repeated a minimum of three times. *Error bars* indicate standard error of the mean. DNA damage foci, comet assay, and qRT-PCR were designed with a minimum of triplicate biological samples. Statistics were determined using GraphPad Prism 8 software. Equal variance and normality were tested to decide which kind of test was run. A two-way ANOVA (α = 0.05) with Dunnett’s multiple comparisons was performed unless otherwise indicated (∗∗, *p* < 0.001; ∗, *p* < 0.05) within each time point or treatment, comparing each column with the corresponding control.

## Data Availability

All data are contained within the article.

## Conflict of interest

The authors declare no conflicts of interest with regard to this article.
